# Intestinal barrier function in response to abundant or depleted mucosal glutathione in *Salmonella*-infected rats

**DOI:** 10.1186/1472-6793-9-6

**Published:** 2009-04-17

**Authors:** Marleen TJ van Ampting, Arjan J Schonewille, Carolien Vink, Robert Jan M Brummer, Roelof van der Meer, Ingeborg MJ Bovee-Oudenhoven

**Affiliations:** 1 TI Food and Nutrition, Wageningen, the Netherlands; 2 Department of Health and Safety, NIZO food research, Ede, the Netherlands; 3 Nutrition and Toxicology Research institute Maastricht, Maastricht University, Maastricht, the Netherlands; 4 School of Health and Medical Sciences, Örebro University, Örebro, Sweden

## Abstract

**Background:**

Glutathione, the main antioxidant of intestinal epithelial cells, is suggested to play an important role in gut barrier function and prevention of inflammation-related oxidative damage as induced by acute bacterial infection. Most studies on intestinal glutathione focus on oxidative stress reduction without considering functional disease outcome. Our aim was to determine whether depletion or maintenance of intestinal glutathione changes susceptibility of rats to *Salmonella* infection and associated inflammation.

Rats were fed a control diet or the same diet supplemented with buthionine sulfoximine (BSO; glutathione depletion) or cystine (glutathione maintenance). Inert chromium ethylenediamine-tetraacetic acid (CrEDTA) was added to the diets to quantify intestinal permeability. At day 4 after oral gavage with *Salmonella enteritidis *(or saline for non-infected controls), *Salmonella* translocation was determined by culturing extra-intestinal organs. Liver and ileal mucosa were collected for analyses of glutathione, inflammation markers and oxidative damage. Faeces was collected to quantify diarrhoea.

**Results:**

Glutathione depletion aggravated ileal inflammation after infection as indicated by increased levels of mucosal myeloperoxidase and interleukin-1β. Remarkably, intestinal permeability and *Salmonella*  translocation were not increased. Cystine supplementation maintained glutathione in the intestinal mucosa but inflammation and oxidative damage were not diminished. Nevertheless, cystine reduced intestinal permeability and *Salmonella* translocation.

**Conclusion:**

Despite increased infection-induced mucosal inflammation upon glutathione depletion, this tripeptide does not play a role in intestinal permeability, bacterial translocation and diarrhoea. On the other hand, cystine enhances gut barrier function by a mechanism unlikely to be related to glutathione.

## Background

During foodborne *Salmonella enteritidis *infection pathogens can pass the gut epithelial cell lining and translocate to extra-intestinal organs like the spleen and liver[1]. In response to mucosal invasion of pathogens, epithelial cells and macrophages express pro-inflammatory cytokines, e.g. interleukin-1β (IL-1β), to recruit neutrophils[2,3]. These efficient killers of translocating microbes [4] contain high concentrations of the enzyme myeloperoxidase (MPO)[4,5], which participates in the innate immune defence through formation of powerful reactive oxidants[4,5]. An unfortunate side-effect of this defence machinery is that the anti-bacterial reactive oxygen species produced also react with cellular organic molecules and have the potential to induce oxidative tissue damage [4-6]. For example, MPO is associated with oxidative stress-related damage (protein nitration) in the inflamed mucosa of ulcerative colitis patients[7]. To reduce this damage, inhibition of MPO has even become a possible target of new drug development[8].

Glutathione, a tripeptide composed of γ-glutamic acid, cysteine and glycine is quantitatively the most important low-molecular-weight (non-protein) thiol in tissues[6,9,10]. Glutathione, synthesised by most mammalian cells but mainly in the liver[9,11], is an active free-radical-scavenging compound found in virtually all animal cells[6,9,11]. In comparison with a number of other tissues the liver has a particularly high content of glutathione[9]. A relatively high concentration of glutathione is also detected in the intestinal epithelium[12]. Here glutathione has been shown to play an important role in the protection of the intestinal mucosa against oxidative stress both in vitro [13,14] and in vivo[12,15]. For example, glutathione depletion in newborn rats leads to increased nitrosative stress during necrotising enterocolitis[15]. Furthermore, *Salmonella* infection decreases enterocyte glutathione levels in mouse ileal loops and this reduction increases the susceptibility of epithelial cells to oxidative damage[16]. This oxidative damage in its turn might impair barrier function. As well as the gut microbiota[17], mucus[18] and the immune system[19,20], intestinal glutathione is suggested to be important for intestinal barrier function[15]. Many studies on the role of glutathione in prevention of oxidative damage in the intestinal mucosa have been performed [13-15]. However, actual in vivo proof that intestinal glutathione is important for gut barrier function is lacking. We therefore investigated the role of glutathione in intestinal barrier function and infection-induced mucosal inflammation.

Buthionine sulfoximine (BSO) is a specific inhibitor of γ-glutamylcysteine synthetase (gamma-GCS), which is the rate-limiting enzyme of glutathione synthesis[21]. This chemical causes glutathione-deficiency in animals[22] and enables us to investigate the role of this tripeptide in animal models. Besides glutathione depletion, stimulation of synthesis is interesting for that purpose as well. Cysteine is known to stimulate glutathione synthesis[23], and cysteine availability is often the limiting factor for intracellular glutathione synthesis[24]. For example, intraperitoneally administered N-acetylcysteine was shown to increase hepatic and intestinal glutathione levels in bile-duct ligated rats[25]. Therefore, dietary supplementation with cysteine, or the more stable variant cystine, can potentially maintain or increase hepatic and intestinal glutathione levels during oxidative stress.

Our aim was to determine whether depletion of glutathione by BSO affects gut barrier function and increases susceptibility of rats to *Salmonella* infection and the associated inflammation. In addition, the effect of dietary cystine on glutathione levels in the intestinal mucosa and consequences for the resistance to infection were investigated.

## Results

### Animals and food intake

At the start of the study, mean body weight of the animals was 243 g. Average food intake before infection was 19 g/d in the control and cystine group, and 17 g/d in the BSO group (p < 0.05). Post infection, food intake was 16 g/d in all groups. Mean body weight gain prior to infection was 5 g/d. After infection, average body weight gain was 3 g/d in all groups.

### BSO decreases the glutathione content in liver and ileum mucosa

**Figure 1:**
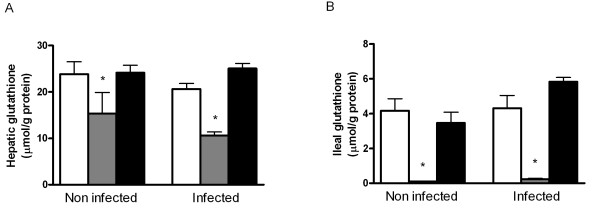
**Total glutathione in liver and ileum mucosa**. Total glutathione in liver (A) and ileum mucosa (B) of rats fed the control diet (white bars) or the same diet supplemented with buthionine sulfoximine (BSO; grey bars) or cystine (black bars). Rats were orally infected with 1.10^9^colony-forming units *S. enteritidis* (n = 8 per diet) or received saline only (non-infected animals; n = 6 per diet). Results are expressed as means ± SE. An asterisk indicates a significant difference from the control diet group (either non-infected or infected rats; p < 0.05).

BSO decreased hepatic glutathione by 48% in the infected animals in comparison with the control group (Figure 1A; p < 0.05). Cystine supplementation did not significantly affect liver glutathione of non-infected rats. Post-infection levels were 21% higher in cystine-fed animals, although this increase did not reach statistical significance. BSO decreased ileal mucosal glutathione by ≥ 98% in non-infected and infected rats (Figure 1B; p < 0.05). Dietary cystine did not increase ileal glutathione in non-infected or infected rats.

BSO is a competitive inhibitor of gamma-GCS and could possibly cause an accumulation of cysteine in the ileum mucosa. However, mucosal cysteine levels were decreased in BSO treated animals (data not shown).

### Diarrhoea, faecal excretion and translocation of *Salmonella*

**Figure 2:**
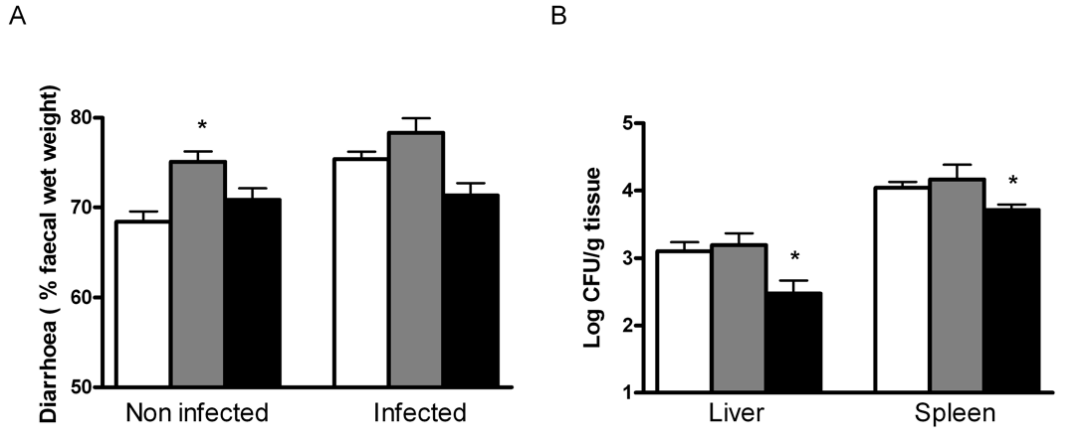
**Relative faecal wet weight and Salmonella translocation**. Relative faecal wet weight (A) before (n = 6 per diet group) and after oral *S. enteritidis* infection (n = 8 per diet group) of rats fed the control diet (white bars) or the same diet supplemented with BSO (grey bars) or cystine (black bars). Translocation of *S. enteritidis *as determined by viable pathogen counts (B) in liver and spleen. Results are expressed as means ± SE. CFU means colony-forming units. An asterisk indicates a significant difference from the control diet group (either non-infected or infected rats; p < 0.05).

Glutathione depletion increased relative faecal wet weight in non-infected animals whereas cystine had no effect (Figure 2A). After infection, there was no difference in diarrhoea between the control and BSO group or between the control and cystine group.

Faecal *Salmonella* excretion was similar in all diet groups on the first and third day after *Salmonella* infection (10^7 ^and 10^6 ^colony-forming units (CFU)/g faeces, respectively), indicating identical intestinal colonisation levels in all groups. Furthermore, depletion of mucosal glutathione did not affect *Salmonella* translocation to liver and spleen (Figure 2B). In contrast, cystine decreased the number of *Salmonella* in both tissues (Figure 2B), pointing to a protective effect of cystine on *Salmonella* translocation to extra-intestinal organs.

### Mucosal antioxidant capacity

**Figure 3:**
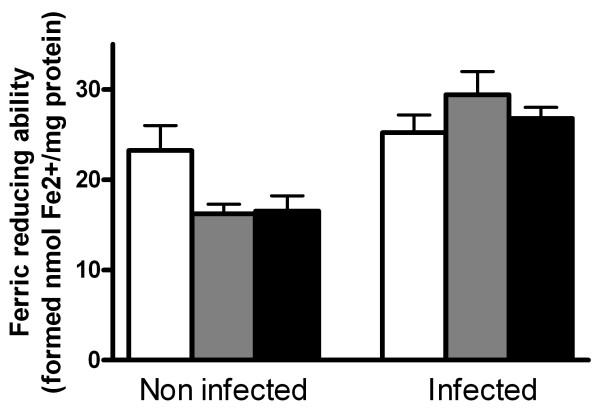
**Ferric reducing ability of non-thiols in the ileum mucosa**. Ferric reducing ability of non-thiols in the ileum mucosa of non-infected rats (n = 6 per diet) and *S. enteritidis* infected rats (n = 8 per diet) fed the control diet (white bars) or the same diet supplemented with BSO (grey bars) or cystine (black bars). All results are expressed as means ± SE. No significant differences were detected between the control diet group and the BSO or cystine-supplemented group.

In response to a serious decrease of glutathione, the body could compensate by increasing other (non-thiol) antioxidants. Therefore we investigated the total mucosal antioxidant capacity of non-thiols by determining the ferric reducing ability of mucosal tissue. Neither BSO nor cystine changed the mucosal non-thiol antioxidant capacity before infection. The same results were found in infected rats (Figure 3).

### Mucosal inflammation and oxidative damage

**Figure 4:**
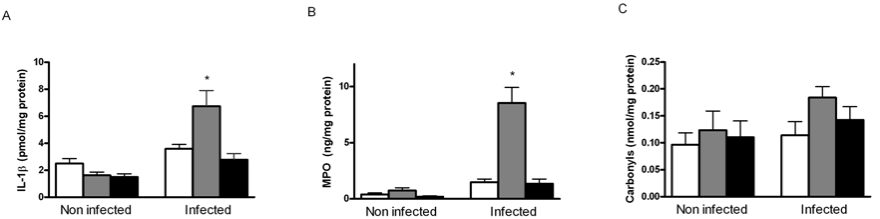
**Inflammation and oxidative damage in the ileummucosa**. BSO increased inflammation in the ileum mucosa of *S. enteritidis *infected rats. *S. enteritidis* infected (n = 8 per diet) and non-infected (n = 6 per diet) rats were fed the control diet (white bars) or the same diet supplemented with BSO (grey bars) or cystine (black bars). Mucosal levels of the pro-inflammatory cytokine interleukin-1β (IL-1β) and myeloperoxidase (MPO) are shown in panels A and B, respectively. Recovery experiments with purified IL-1β and MPO, added to the mucosal samples, were included in each assay and confirmed the absence of inhibiting factors in the ileal samples. Oxidative damage to ileal proteins was determined by measurement of protein carbonyls (C). All results are expressed as means ± SE. An asterisk indicates a significant difference from the control diet group (either non-infected or infected rats; p < 0.05).

Despite identical colonisation levels, the glutathione-depleted rats, presumably with lower antioxidant capacity, had increased ileal mucosal IL-1β levels (Figure 4A). This coincided with a more intensive inflammatory response in this group as measured by ileal MPO (Figure 4B). However, this did not increase oxidative-stress related mucosal damage like protein carbonyls (Figure 4C) and mucosal lipid peroxidation, measured by thiobarbituric acid reactive substances (TBARS; results not shown). Despite protective effects of cystine on *Salmonella* translocation and maintenance of glutathione levels, intestinal inflammation and oxidative damage markers were not decreased in this group (Figure 4).

### Intestinal permeability

**Figure 5:**
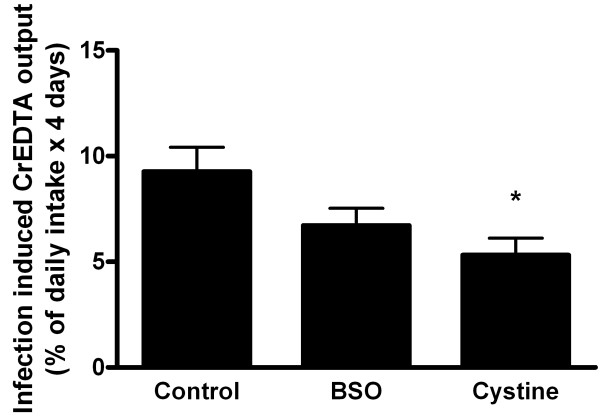
**Infection-induced urinary excretion of dietary CrEDTA**. Infection-induced urinary excretion of dietary CrEDTA after oral *S. enteritidis* infection (n = 8 per diet) in rats fed the control diet or the same diet supplemented with BSO or cystine. Results, expressed as means ± SE, were corrected for baseline (pre infection) permeability. Baseline CrEDTA output was identical in all groups. The asterisk indicates a significant difference from the control group (p < 0.05).

In general, the *Salmonella* infection increased paracellular intestinal permeability with time (Figure 5). Despite increased mucosal inflammation in BSO treated animals, intestinal permeability was not aggravated. In contrast, the results suggest a decrease in comparison with the control group (Figure 5). Moreover, cystine partially restored infection-induced intestinal permeability to basal (pre-infection) levels, suggesting a beneficial effect of cystine on gut barrier function. Neither BSO nor cystine supplementation affected intestinal permeability in non-infected rats (data not shown).

### Nitric oxide response is not affected by the cystine diet

Since diet composition can affect the inducible nitric oxide (NO) production capacity in response to microbial stimuli, the effect of dietary cystine was investigated in an LPS experiment. Urinary NO_x _(sum of nitrate and nitrite) excretion in response to intraperitoneally administered lipopolysaccharide (LPS) was identical in the control and cystine group (data not shown), demonstrating equal host capacity to produce NO.

## Discussion

The present study shows that depletion of glutathione during *Salmonella* infection results in increased mucosal inflammation. However, as *Salmonella* translocation was not affected by BSO treatment, glutathione depletion obviously does not affect mucosal barrier function. Also, urinary excretion of the paracellular permeability marker chromium ethylenediamine-tetraacetic acid (CrEDTA) was not increased in comparison with infected rats fed the control diet. On the other hand, cystine supplementation resulted in improved barrier function and decreased *Salmonella* counts in extra-intestinal organs. However, the subsequent ileal inflammation was not reduced. Since cystine did not significantly increase glutathione and inflammation was not reduced the results suggest that the protective mechanism of cystine supplementation is not related to mucosal glutathione levels. Most likely other host defences than glutathione are strengthened by cystine.

In line with other studies, oral administration of BSO, a selective inhibitor of glutathione synthesis, decreased hepatic glutathione levels[26,27]. Nearly complete depletion was observed in ileal mucosa of both infected and non-infected animals. A decrease of intestinal glutathione is shown during necrotising enterocolitis in rats[15] and in mouse ileal loops exposed to *Salmonella typhimurium*[16]. This decrease is suggested to contribute to pathogenesis of disease and subsequent failure of barrier integrity[15,16]. Our results now show that depletion of glutathione does not necessarily lead to impaired intestinal barrier function. A study with mice indicated that glutathione-depletion by BSO caused mucosal damage in the jejunum and colon[28]. However, a much higher dose of BSO was applied, increasing the risk of pharmacological side-effects of BSO in that study. The lower dose used in our study, nearly depleted intestinal mucosal glutathione but hardly affected body weight gain and did not result in histological mucosal damage in haematoxylin- and eosin-stained ileum sections (data not shown).

The oral *Salmonella* infection in our experiment had no or little effect on liver and ileal mucosal glutathione at 4 days post infection, indicating that the organ-specific capacity to synthesise glutathione was adequate or that infection-induced oxidative stress was absent in normally fed rats (diet adequate in antioxidants). In previous studies we (unpublished results) and others[15] detected a temporarily decrease of intestinal glutathione after an intestinal insult. So, intestinal glutathione levels may differ depending on the time point studied, which may result in seemingly contrasting results. For example, in our in-vivo study, glutathione was determined at 4 days after infection and levels were not (or no longer) decreased. However, a reduction in glutathione was reported in an ileal loop study at 18 hrs after *Salmonella* introduction [16]. Furthermore, studies limited to specific cell types, for example enterocytes isolated from infected animals[16], may show different results from in vivo studies investigating complete mucosa. Specifically, during infection T lymphocytes and macrophages migrate into the intestinal mucosa and these cells are also able to synthesise glutathione and thus contribute to mucosal glutathione [23,29]. In our study, oxidative damage in the form of intestinal protein carbonyls or TBARS was not detected when intestinal glutathione was depleted (BSO treated rats). Oxidative damage induced by glutathione depletion has been shown in plasma lipids [30] and liver (DNA adducts)[26], however those studies did not investigate the intestine.

Supplementation of cystine maintained intestinal glutathione levels similar to the control diet group. Surprisingly, inflammation was not reduced despite a decrease in intestinal permeability and significant inhibition of *Salmonella* translocation. Improvement of gut barrier function has also been described for intraperitoneally administered N-acetylcysteine in partially hepatectomised rats[31]. The reduced viable *Salmonella* counts in liver and spleen of the cystine-fed rats in our study may have resulted from decreased *Salmonella* translocation from the gut or from enhanced killing of translocated pathogens by the innate immune system, *e.g. *by increased production of nitric oxide (NO)[32,33]. N-acetylcysteine can enhance this NO defence[34], but the effect of cystine was unknown. Our LPS experiment revealed that rats fed the control and cystine diet responded identically to an intraperitoneal LPS challenge, excluding differences in the NO-based capacity of the host to kill bacterial invaders. Therefore, the lower *Salmonella* counts in liver and spleen observed here probably reflect reduced translocation of this pathogen from the gut lumen.

Surprisingly, infection-induced intestinal permeability of BSO-treated animals seemed lower than that of the control group (Figure 5) but the effect was not significant. One could hypothesize that competitive inhibition of gamma-GCS may cause accumulation of cysteine in the mucosa, resulting in protective effects as seen on the cystine diet. However, mucosal cysteine levels were decreased in BSO treated animals. Furthermore, in contrast to the cystine fed rats, no protective effect against *Salmonella* translocation was observed in these animals.

The mucosal barrier enhancing effect of cystine requires further investigation. Results need to be reproduced and the mechanism behind the protective effect should be explored. Cysteine can play a role in various host defences. For instance, trefoil peptides, which stabilise mucosal glycoproteins and protect the mucosa from various insults, e g bacterial toxins[35], contain several cysteine-rich domains[35,36]. In addition, defensins, which are antimicrobial peptides secreted by human and rodent ileal Paneth cells, contain 6 conserved cysteines[37] and are important for intestinal resistance to *Salmonella* infection[38]. Although, cysteine is not an essential amino acid and can be synthesised from methionine, it might be conditionally essential like suggested for glutamine[39,40].

## Conclusion

In conclusion, intestinal depletion of glutathione is not as detrimental for maintenance of the gut barrier as often presumed. Neither paracellular intestinal permeability nor *Salmonella* translocation were promoted at very low mucosal glutathione levels, despite increased infection-induced ileal inflammation. On the other hand, dietary cystine did not reduce infection-associated inflammation but decreased intestinal permeability and *Salmonella* translocation to extra-intestinal organs by a mechanism unlikely related to glutathione. Follow-up studies are needed to elucidate the molecular mechanism(s) underlying the protective effect of cystine supplementation on gut barrier function and to address human relevance.

## Methods

### Diets, infection and dissection of the rats

The animal experiments were approved by the animal welfare committee of Wageningen University (Wageningen, the Netherlands). Specific-pathogen-free male Wistar rats (WU, Harlan, Horst, the Netherlands), 8 weeks old at the start of the dietary intervention, were housed individually in metabolic cages. Rooms were temperature (20–21°C) and humidity-controlled (50–60%) with a 12:12-hour light/dark cycle. Rats were fed purified diets containing per kg: 200 g acid casein, 326 g cornstarch, 174 g glucose, 160 g palm oil, 40 g corn oil, 50 g cellulose and vitamin and mineral mix (without calcium) according to AIN-93[41]. To mimic the composition of a Western human diet, the prepared diets were relatively low in calcium (30 mmol/kg) and high in fat content (200 g/kg) in comparison with recommendations for rodent diets by AIN-93. This control diet was supplemented with DL-buthionine(*S*, *R*)-sulfoximine (BSO) or L-cystine (both purchased from Sigma-Aldrich, St. Louis, Missouri, USA), at the expense of glucose, to a final concentration of 7.5 mmol/kg and 12.3 mmol/kg, respectively. Nitrogen correction of the diets was considered unnecessary, since the contribution of cystine was marginal (0.035%). In addition, the inert intestinal permeability marker CrEDTA was added to the diets[42,43]. Preparation and purity control of CrEDTA was performed as described [44-46]. The CrEDTA solution was lyophilised in a manifold freeze dryer (FD5515; Ilshin Laboratory Co Ltd, Seoul, South Korea) and the dry material obtained was added to the diets to a final concentration of 2 g/kg.

Food and demineralised drinking water were supplied ad libitum. Body weight and food intake were recorded twice a week before infection and daily after infection. One group was fed the control diet and another group was fed the cystine-supplemented diet (n = 14 per diet). The third group (n = 14) was fed the control diet for one week, followed by the BSO diet for the remaining study period.

Animals were acclimatised to housing and dietary conditions for 21 days, after which 8 animals per diet group were orally infected with 1 ml of saline containing 1.10^9 ^CFU of *Salmonella* enteritidis(clinical isolate, phage type 4; strain B1214 NIZO food research, Ede, the Netherlands) as described elsewhere[47]. The other 6 animals per diet group were not infected and received 1 ml saline orally.

In previous *Salmonella* infection studies in rats we have found that levels of ileal mucosal glutathione change with time (unpublished). After an initial decrease, total glutathione levels start to recover at day 4 post infection. Furthermore, for monitoring functional infection outcomes like *Salmonella* colonisation, translocation and infection-induced permeability changes, follow-up of infected rats for at least 3 to 4 days is needed. Therefore section was performed at day 4 after infection (or sham treatment). Rats were randomly selected and killed by carbon dioxide inhalation. During the dissection, the distal 12 cm of the ileum was excised and cut open longitudinally. After flushing rapidly with saline, the mucosa was scraped off with a spatula and immediately frozen in liquid nitrogen for protein analyses. Furthermore, the spleen and liver were aseptically excised and after homogenisation in sterile saline directly used for *Salmonella* quantification, as described elsewhere[48]. Another part of the liver was immediately frozen in liquid nitrogen for further preparation and analyses as described below.

Fresh faecal samples were collected to quantify *Salmonella* colonisation prior to infection and 1 and 3 days post infection, as described elsewhere[49]. In addition, 24h faeces (pooled per animal in both the pre-infection and post-infection period) and urines were collected from 2 days before infection until 4 days after oral *Salmonella* administration. All faeces and urine samples were stored at -20°C until further analysis. Oxytetracycline (Sigma-Aldrich) was added to the urine collection vessels of the metabolic cages to prevent bacterial deterioration.

### *Salmonella*colonisation and diarrhoea

Faecal *Salmonella* was determined in fresh faecal samples collected with time as described elsewhere[49]. Total 24 h faeces were lyophilized in a manifold freeze dryer (FD5515; Ilshin Laboratory Co Ltd). Faecal water was prepared as described previously and osmolarity was measured (Osmomat 030-D, Gonotec, Berlin, Germany) to calculate the percentage wet weight[50]. Direct determination of faecal wet weight by lyophilisation was considered inappropriate because this underestimates relative faecal wet weight due to evaporation of water from the faecal pellets in the collection vessels of the metabolic cages.

### Intestinal permeability analysis

CrEDTA excreted in 24 h urine samples was determined by inductively coupled plasma atomic emission spectroscopy (ICP-AES; Varian, Mulgrave, Australia) as described elsewhere[45]. Urinary CrEDTA output was expressed as percentage of dietary intake. Infection-induced urinary excretion of CrEDTA was corrected for baseline (pre-infection) levels. Urine was tested negative for faecal contamination by checking the absorption spectrum 400–600 nm for bile pigments. These pigments are present in faeces but absent in urine.

### Tissue sample preparation

Frozen liver tissue and ileal mucosa were pulverised under liquid nitrogen. Frozen pulverised tissue was suspended in a 0.2 M sucrose buffer of pH 7.4 containing 20 mM trishydroxymethylaminomethane (Tris), 1 mM dithiothreitol (DTT; Sigma Aldrich) and Complete Protease Inhibitor Cocktail (Roche Diagnostics, Basel, Switzerland) and sonicated on ice for 20–30 s at level 2–3 (Sonicator XL2020, Heat Systems, Farmingdale, New York, USA). The protein concentration of the samples was determined using biocinchoninic acid (BC) Assay (Omnilabo, Veenendaal, the Netherlands).

### Total glutathione and cysteine analyses

Total glutathione in liver and ileum was measured using a slightly modified assay described by Mansoor *et al*[51]. This method determines the total of reduced, oxidized, and protein-bound forms of glutathione by HPLC without discrimination of the individual forms. As a result, total glutathione levels are presented and indicated in the text by 'glutathione'. Compared to the original method, twice the sample volumes and reagents were used. Briefly, samples of 25 μl were injected into a 150 × 4.6 mm, 3 μm PLRP-S column, equipped with a PLRP-S guard column (Polymer Laboratories, Amherst, Massachuessetts, USA). Flow rate was 1 ml/min at 30°C with elution solvent A (0.1% trifluoric acid (TFA), 5% acetonitrile) and solvent B (0.1% TFA, 80% acetonitrile), both diluted with distilled water. Chemicals were purchased from Sigma-Aldrich. The elution profile was as follows: 0–20 min, 0% B; 20–25 min, 16% B; 25–30 min 50% B, with retention time of bimane derivatives of glutathione of 23 min. A Spectra Systems FL2000 fluorometer (Spectra Physics, Mountain View, California, USA) was used for detection, with excitation and emission at 394 and 480 nm, respectively. Plotting and integration of peaks were performed by Chromeleon software 6.6 (Dionex, Sunnyvale, California, USA). Total glutathione results are expressed as μmol/g tissue protein.

In the samples described above cysteine, identified as a separated peak in chromatograms, was determined identically and simultaneously with the glutathione analysis.

### Mucosal antioxidant capacity

Mucosal antioxidant capacity of non-thiols, thus excluding glutathione, was determined using the ferric reducing ability method as described for plasma[52]. Briefly, to 33 μl ileum sample 1 ml of ferric reducing ability reagent was added[52]. Antioxidant capacity is expressed as nmol Fe^2+ ^formed per mg protein.

### Mucosal inflammation and oxidative damage

A mouse MPO ELISA test kit (Hycult biotechnology, Uden, the Netherlands), which is cross-reactive with rat MPO, was used to determine the concentration of MPO in mucosal scrapings. IL-1β levels were determined using an ELISA kit (Biosource, Nivilles, Belgium).

To evaluate lipid peroxidation, TBARS in the mucosal samples were determined according to Ohkawa[53], using 100 μl of undiluted sample[50]. Control experiments with reference samples, known to have TBARS, were analysed positive. In addition, spiking of ileal mucosal samples of the present study with the reference material showed good recovery of TBARS.

Ileal protein carbonyls, as marker of protein oxidation, were measured by an ELISA according to the method of Buss *et al.*[54]. Briefly, standards and samples were diluted in phosphate-buffered saline to a protein concentration of 4 mg/ml. Carbonyls were derivatised with 2,4-dinitrophenyl hydrazine (Sigma-Aldrich) and coated on an ELISA plate (Nunc-Immuno plate maxisorp, Nunc, Roskilde, Denmark). After probing with biotinylated anti-2,4-dinitrophenyl hydrazine antibody (Molecular Probes Inc, Eugene, Oregon, USA), streptavidin-biotinylated horseradish peroxidase (Amersham Biosciences, Piscataway, New Jersey, USA) was incubated in the wells. Staining was performed with *o*-phenylenediamine (Sigma-Aldrich). Absorbance was read at 490 nm in a ThermoMax microplate reader (Molecular Devices Corp, Sunnyvale, California, USA).

### *S. enteritidis *lipopolysaccharide experiment

To study the effect of dietary cystine on the capacity of rats to generate NO in response to systemic bacterial stimuli, rats (n = 8 per group) fed the control or cystine diet (dietary acclimatisation of 14 days) were intraperitoneally injected with 0.5 mg/kg LPS, as described elsewhere [48]. The LPS used was derived from *S. enterica *Serovar enteritidis, which is identical to the strain used in the oral infection study. Animals were monitored until 3 days after challenge and 24 h urines were collected. The NO response was quantified by measuring urinary NO_x _(sum of nitrate and nitrite) excretion by using a colorimetric enzymatic kit (Roche Diagnostics), as described elsewhere[55].

### Statistical analysis

All data are expressed as means ± SE. Data were tested for normality by the Kolmogorov-Smirnov test. If normally distributed, differences between the control and dietary intervention groups (cystine or BSO) were tested for significance using one-way ANOVA, followed by Bonferroni's multiple-comparison test. Our aim was to investigate dietary effects in the non-infection and the infection period separately. Therefore, differences between non-infected and infected groups were not tested. When data of groups showed unequal variances, the Kruskal-Wallis test was used, followed by Dunn's post test for multiple comparisons. Statistical significance was set at p < 0.05.

## Competing interests

The authors declare that they have no competing interests.

## Authors' contributions

MvA, RM and IB designed the study. MvA, AS and CV performed the experimental work. MvA, RvdM and IB analysed the data. MTJA and IB wrote the manuscript and RvdM plus RB provided valuable feedback on the initial draft. All authors read and approved the final manuscript.
